# Clinical Study of a New Modified Early Warning Scoring System for Rapidly Evaluating Shock in Adults

**DOI:** 10.7759/cureus.38224

**Published:** 2023-04-27

**Authors:** Rohith N, Srikanth Narayanaswamy, Swati Hegde

**Affiliations:** 1 Internal Medicine, MS Ramaiah Medical College, Bengaluru, IND

**Keywords:** intensive care unit, mews score, apache 2 score, sofa score, shock

## Abstract

Background: Shock is one of the most common severe syndromes requiring emergency treatment. Acute myocardial infarction guidelines, the surviving sepsis campaign, and low blood volume resuscitation guidelines indicate the prioritization of early identification of shock. APACHE II (Acute Physiology and Chronic Health Evaluation II), SOFA (Sequential Organ Failure Assessment), and MEWS (Modified Early Warning System) scores are used to predict mortality in ICU (intensive care unit) patients. However, similar to APACHE II, SOFA cannot be used for rapid assessment. Hence the need for a new scoring system that is simple, faster, and efficacious in predicting and preventing mortality among shock patients. The present study was conducted to evaluate a new MEWS scoring system for early identification and estimate mortality risk in patients with shock.

Methods: A total of 170 patients with shock meeting the inclusion criteria who presented to the ICU of Ramaiah Hospitals from November 2019 to August 2021 were included in the study. Baseline variables, laboratory parameters, APACHE II score, SOFA score, MEWS score, and new MEWS score were compared between the two groups. Patients were followed up till mortality or discharge from ICU.

Results: Among the 170 patients included in the study septic shock (69.4%) was the most common type of shock followed by cardiogenic (7.64%) and hypovolemic shock (7.64%). The requirement of inotropic support and mechanical ventilation was associated with significantly higher mortality (55.6% and 52.6% respectively). The AUROC (area under the curve) for SOFA in predicting mortality was 0.738 (p<0.001). The AUROC for APACHE II score in predicting mortality was 0.758 (p<0.001). The AUROC for MEWS score in predicting mortality was 0.655 (p=0.0002). The AUROC for the new MEWS score in predicting mortality was 0.684 which is more than that of the conventional MEWS score (p <0.001).

Conclusion: New MEWS score is better than the MEWS score in predicting mortality. The new MEWS score is a simple, entirely clinical, inexpensive scoring system that can be done within a short time as a patient contact in an emergency. Hence, the new MEWS score can help in the quick identification of patients who are at high risk for developing shock and can be used as a better predictor of mortality.

## Introduction

Shock is a life-threatening condition of hemodynamic circulatory failure causing inadequate perfusion to meet cellular and metabolic needs. The consequences of shock can be reversible and irreversible which progress to MODS (Multiple Organ Dysfunction Syndrome) and mortality. Septic shock is among the most common type of shock in ICU (intensive care unit) patients. This is followed by cardiogenic and hypovolemic shock [1,2]. In a clinical trial involving 1600 patients with shock, 62% had septic shock, 16% had cardiogenic shock, 4% had other types of distributive shock (anaphylaxis, neurogenic), and 2% had obstructive shock [2]. About 5.3 million people, around the world, perish due to sepsis every year [3]. At the start of the 21st century, the incidence of sepsis increased and the mortality decreased [4]. Sepsis is more common in men than women and especially in those with age more than 65 years [3,5].

Although there are currently no screening tests that can assess high-risk patients who develop shock. In addition, diagnostic tests for sepsis (e.g. culture) take at least 48 hours to be positive, and delays in the use of antibiotics can lead to worse outcomes. Therefore, clinicians must rely on their clinical judgment to suspect sepsis in these patients of shock. There have been several attempts to develop bedside scoring systems using simple clinical criteria to potentially help physicians identify patients with sepsis or patients who may develop shock. Some commonly used outcome predictors are the SOFA (Sequential Organ Failure Assessment) score, APACHE (Acute Physiology and Chronic Health Evaluation) II score, and MEWS (Modified Early Warning System) score.

Some drawbacks of the APACHE II score are that it is complicated and burdensome to use. Its predictive value at 24 hours is very poor [6]. APACHE II was studied on patients directly admitted to the ICU, it is not accurate when being used on patients transferred from wards or another hospital. This can be used as a triage tool and to predict the treatment response on admission. SOFA score uses objective, readily available measures to quantify the dysfunction of six organ systems. The dysfunction of each organ is rated according to a scale (0 (normal function) to 4 (organ failure)), and individual scores can then be summed to provide a total. SOFA scores correlate with mortality. A decreasing trend of SOFA score during the first 48 hours is associated with a decrease in mortality rates [7]. MEWS can be used to quickly identify clinical deterioration and determine whether a higher level of care is necessary for all hospital patients, regardless of whether they are in a ward or an ICU. Lower MEWS patients can still receive routine care and be monitored. Patients with high MEWS should receive close monitoring and consideration should be given to transferring them to a higher level of care, such as an ICU.

While Naeem and Montenegro showed that the MEWS has a limited capacity to estimate sudden deterioration in patients with cardiogenic shock, Subbe et al. reported that the MEWS has a poor resolving ability for those patients in shock without any symptoms in an accident and emergency situations [8,9]. Age, body temperature, heart rate, respiratory rate, blood pressure, pulse oximetry, and state of consciousness were chosen as indicators based on the accessibility and timeliness features of early rapid prediction of emergency situations. Oxygen saturation is also important and related to the prognosis of patients in shock and is easily obtained.

Heart rate, respiratory rate, temperature, systolic pressure, age, oxygen saturation, and consciousness index are all included in the new MEWS scoring system, and all of these indicators may have similar indices to those in the APACHE II [10]. In the study conducted by Qin et al., a new MEWS system was created which includes all the parameters of the MEWS system together with transcutaneous oxygen saturation [10]. Based on this background, we intended to compare APACHE II, SOFA, MEWS, and new MEWS in predicting mortality among shock patients in ICU and validate a new MEWS scoring system for early identification and estimate mortality risk in patients with shock.

## Materials and methods

A hospital-based prospective study of adult patients with shock admitted to the ICU in Ramaiah Hospital was conducted. The prospective data collection was done over one year and 10 months (October 2019 to August 2021). The study included either patients with sepsis admitted from the emergency room or those who developed sepsis during hospitalization. Patients admitted to ICU from the emergency room and those re-admitted during the observation period were not included. A total of 170 patients with shock were admitted to the ICU, the purpose of the study was explained and informed consent was taken from the health care proxy. This study was done after the ethical committee approval of MS Ramaiah Medical College and Hospital with vide letter no. “MSRMC/EC/AP-18/10-2019 dated 12.10.2019”. In the end local, legal and ethical requirements were met with this approval.

Data collected for the study were demographic details, vitals, and investigations including complete blood count, liver function test, renal function test, arterial blood gas, serum electrolytes, and blood/urine/wound culture. Consciousness was assessed using the Glasgow coma scale and AVPU (Alert, Voice, Pain, Unresponsive) scale. The need for inotropes, vasopressors, and ventilator requirements was noted. The ventilation includes both invasive and non-invasive. Data were collected only at the time of ICU admission.

Septic shock, hypovolemic shock, cardiogenic shock, anaphylactic shock, and mixed shock, which include two or more of the aforementioned types of shock, were all diagnosed and categorized in patients. Patients less than 18 years and pregnant women were excluded from the study. Patients were divided into two groups based on mortality: survived and dead. Patients were followed up till the endpoint, which is either mortality or discharge from the hospital.

In the current study, descriptive and inferential statistical analysis was completed. Results for categorical measurements are presented as numbers (%) and results for continuous measurements are presented as mean SD (minimum-maximum). The 5% level of significance is used to determine significance. To determine the significance of study parameters on a continuous scale between two groups (intergroup analysis), a student t-test (two-tailed, independent) was used. The significance of study parameters on a categorical scale between two or more groups was determined using the Chi-square/Fisher Exact test in a non-parametric setting for the analysis of qualitative data. APACHE II, SOFA, MEWS, and new MEWS were compared among the two groups studied. Receiver operating curves were employed to predict mortality using APACHE II, SOFA, MEWS, and new MEWS scores.

## Results

**Figure 1:**
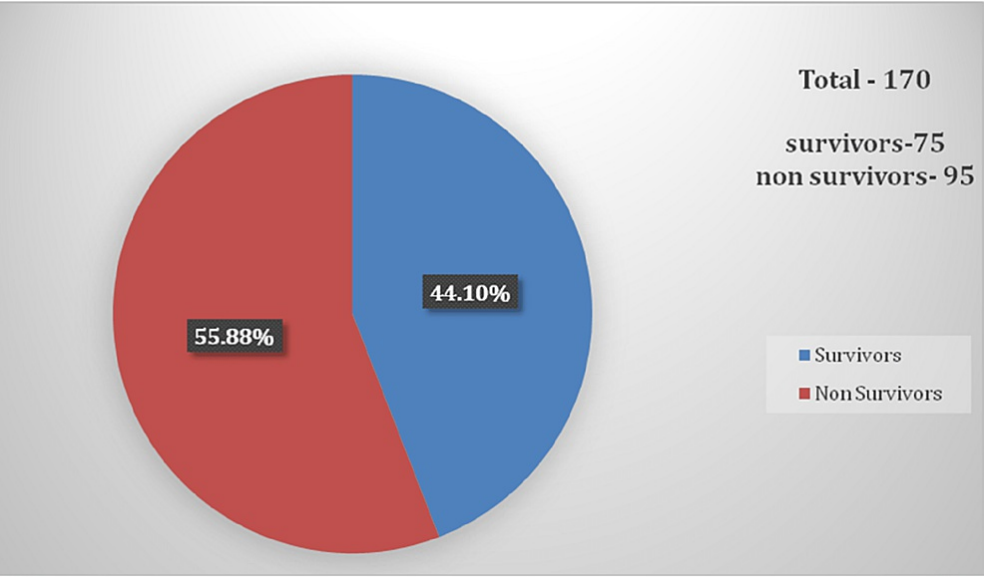
Comparison between the number of survivors and non-survivors in the study

A total of 170 patients were admitted with shock in the ICU of MS Ramaiah Hospital, 75 patients survived (44.1%), and 95 patients died (55.88%) (Figure 1). The patient's average age was 58.22±15.52 years. The majority of the patients were older than 60 years of age (Table 1). Our study revealed that septic shock (69.4%) was the most prevalent type of shock, followed by cardiogenic shock (7.54%) and hypovolemic shock (7.54%) (Table 2).

**Table 1:** Age distribution Mean ± SD: mean ± standard deviation

Age (in Years)	Number of Patients (Percentage)	Mean±SD
<30	10 (5.9)	58.22±15.52
30-40	13 (7.6)	
41-50	25 (14.7)	
51-60	45 (26.5)	
>60	77 (45.3)	
Total	170 (100)	

**Table 2:** Comparison of the types of shock

Type of Shock	Mortality		Total
	Survived	Death	
Septic	49	69	118
Cardiogenic	6	7	13
Hypovolemic	7	5	13
Anaphylactic	2	0	2
Mixed	12	13	25

Table 3 depicts the baseline values of the survivor and non-survivor groups. On comparing age, blood pressure, temperature, and respiratory rate no statistical significance was observed. However, the mean oxygen saturation in survivor and non-survivor groups was 92.12±7.59 and 88.29±9.75 respectively (p=0.005). Moreover, the baseline mean pulse rate also revealed statistical significance between the groups (p=0.024).

**Table 3:** Comparison of baseline variables SBP: systolic blood pressure; DBP: diastolic blood pressure #Data depicted as Mean±SD *p<0.05

Variables	Survivor Group	Non-Survivor Group	Total	P Value
Mean age (in years)	56.73±15.84	59.4±15.25	58.22±15.52	0.267
Mean heart rate (beats per minute)	98.53±18.07	105.28±20.14	102.31±19.49	0.024*
Mean SBP (in mm Hg)	83.31±13.5	82.53±16.65	82.87±15.3	0.742
Mean DBP (in mm Hg)	53.47±13.14	53.72±11.95	53.61±12.45	0.897
Mean arterial pressure (in mm Hg)	62.4±14.78	63.07±11.69	62.78±13.1	0.740
Mean temperature (in Fahrenheit)	98.84±0.97	98.91±1.05	98.88±1.01	0.661
Mean respiratory rate (breaths per minute)	24.95±6.41	25.83±5.97	25.44±6.17	0.354
Mean oxygen saturation (in percentage)	92.12±7.59	88.29±9.75	90.04±9.04	0.005*

On comparing the acid blood gas analysis between the groups, the pH level in the non-survivor group (7.20±0.2) was significantly lower than the survivor group (7.320±0.17) (p<0.001) (Table 4). However, statistically insignificant results were obtained by comparing the partial pressure of oxygen and carbon dioxide between the groups (p>0.05). The ratio between paO2/FiO2 (partial pressure of oxygen/fraction of inspired oxygen) revealed statistically significant results (p<0.001) (Table 4). However, the mean alveolar-arterial gradient in the survivor group was 443.45±140.28, and the non-survivor group was 279.75±198.14 (p<0.001) (Table 4).

**Table 4:** Comparison of mean ABG variables between the two groups paO2: partial pressure of oxygen; pCO2: partial pressure of carbon dioxide; pH: potential of hydrogen; HCO3: bicarbonate; FiO2: fraction of inspired oxygen; A-a gradient: alveolar-arterial gradient; ABG: arterial blood gas #Data depicted as Mean±SD *p<0.05

Variables	Survivor Group	Non-Survivor Group	Total	P Value
paO2	87.01±24.81	84.89±24.85	85.82±24.78	0.58
pCO2	35.71±6.31	33.17±10.33	34.29±8.85	0.07
pH	7.32±0.17	7.2±0.2	7.26±0.2	<0.001*
HCO3	18.96±4.86	15.43±6.09	16.99±5.84	<0.001*
Lactate	3.81±3.03	4.6±3.68	4.25±3.42	0.14
paO2/FiO2	380.82±154.65	279.75±198.14	324.34±186.66	<0.001*
A-a gradient	443.45±140.28	518.72±134.35	504.2±137.53	0.11

Inotropic support (including vasopressors) was provided to 167 patients, 44.3% of the patients belonged to the survivor group and 55.6% to the non-survivor group (p<0.001) (Table 5). Similarly, statistical significance was observed between the two groups regarding the requirement of ventilatory support (p<0.001) (Table 5).

**Table 5:** Comparison of inotropes and ventilator requirement among the patients studied #Data depicted as number (percentage) *p<0.05

Variables		Survivor Group	Non-Survivor Group	Total	P Value
Inotropes	Not Required	1 (33.3)	2 (66.6)	3 (1.8)	<0.001*
	Required	74 (44.3)	93 (55.6)	167 (98.2)	
Ventilator	Not Required	64 (58.7)	45 (41.2)	109 (64.1)	<0.001*
	Required	11 (18.03)	50 (81.97)	61 (35.9)	
Total		75 (44.1)	95 (55.8)	170 (100)	

Table 6 depicts that the mean SOFA score was higher among non-survivors (9.53±4.04) as compared with survivors (6.27±2.98) (p<0.001). Similar statistical significance was observed on comparing the mean APACHE II, mean MEWS, and mean new MEWS score and was found to be higher among non-survivors as compared with the survivors (p<0.001) (Table 6).

**Table 6:** Comparison of mean SOFA, APACHE II, MEWS, and new MEWS scores SOFA: Sequential Organ Failure Assessment; APACHE II: Acute Physiology and Chronic Health Evaluation; MEWS: Modified Early Warning Score #Data depicted as Mean±SD *p<0.05

Variables	Survivor Group	Non-Survivor Group	Total	P Value
SOFA	6.27±2.98	9.53±4.04	8.09±3.95	<0.001*
APACHE II	14.52±6.46	22.06±8.21	18.74±8.36	<0.001*
MEWS	4.92±1.96	6.24±2.31	5.66±2.26	<0.001*
New MEWS	7.48±2.65	9.53±3.43	8.62±3.26	<0.001*

**Figure 2:**
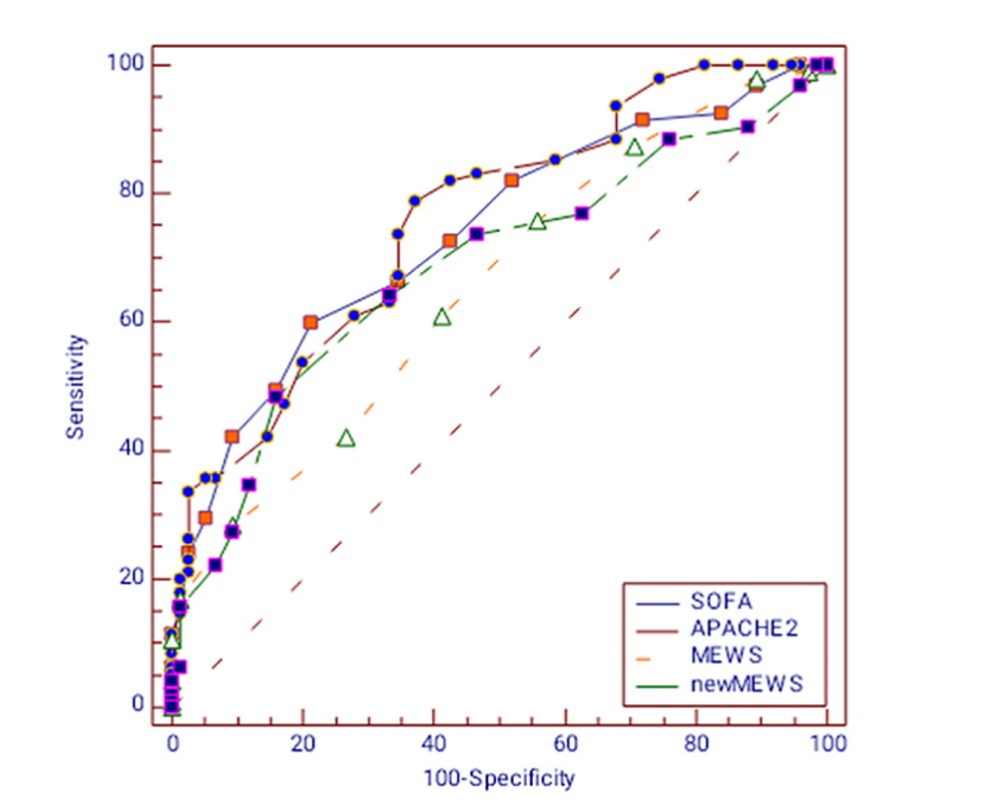
ROC curve for SOFA, APACHE II, MEWS, and new MEWS SOFA: Sequential Organ Failure Assessment; APACHE II: Acute Physiology and Chronic Health Evaluation; MEWS: Modified Early Warning Score; ROC curve: receiver operating characteristic curve

On comparing the ROC curve analysis, the APACHE II had the highest sensitivity of 78.95% compared to other scoring systems (Table 7). However, the new MEWS was found to be the most specific (84%) scoring system (Table 7). The AUROC for new MEWS was 0.684 (p<0.001) (Figure 2). This was statistically significant to MEWS score for which AUROC was 0.655 (p=0.0002), whereas AUROC for SOFA and APACHE II were 0.738 (p<0.001) and 0.758 (p<0.001) respectively (Table 7, Figure 2).

**Table 7:** Comparison of ROC curve analysis to predict mortality SOFA: Sequential Organ Failure Assessment; APACHE II: Acute Physiology and Chronic Health Evaluation; MEWS: Modified Early Warning Score; ROC curve: receiver operating characteristic curve; LR: likelihood ratio; AUROC: area under the ROC curve; SE: standard error *p<0.05

Variables	ROC Results to Predict Mortality				Cut-off	AUROC	SE	P value
	Sensitivity (in percentage)	Specificity (in percentage)	LR+	LR-				
SOFA	60.00	78.67	2.81	0.51	>8	0.738	0.0375	<0.001*
APACHE II	78.95	62.67	2.11	0.34	>15	0.758	0.0364	<0.001*
MEWS	75.79	44.00	1.35	0.55	>4	0.655	0.0413	0.0002*
New MEWS	48.42	84.00	3.03	0.61	>9	0.684	0.0437	<0.001*

## Discussion

One of the more frequently encountered severe syndromes in emergency care is shock. The early detection of shock should be prioritized, according to acute myocardial infarction guidelines, the surviving sepsis campaign, and low blood volume resuscitation guidelines [11-13]. Due to the lack of a universally accepted and widely used specific scoring system, emergency teams typically use the non-specific APACHE II system or MEWS to identify shock patients.

The APACHE II system is effective at determining shock severity and has a higher predictive mortality accuracy for ICU patients, but it requires the collection of more than 10 different types of clinical data, some of which are time-consuming. Higher specificity and accuracy are provided by the SOFA scoring system, which is used to identify patients with MODS. However, the SOFA cannot be used for a quick assessment, just like APACHE II. Therefore, developing a quick scoring system for use in emergency assessments is crucial.

Due to its simplicity and ease of use, MEWS has been the most frequently used scoring system in emergency situations. Burch et al. advocated the implementation of MEWS as its simple indices are helpful in the quick assessment of patients admitted to the ICU (Table 8) [14]. While Naeem and Montenegro demonstrated that the MEWS has a limited capacity to estimate sudden deterioration in patients with cardiac shock, Subbe et al. reported that the MEWS has a poor resolving ability for those patients in shock without any symptoms in accidental and emergency situations [9,15].

**Table 8:** New MEWS scoring system [10] MEWS: Modified Early Warning System

Scores	3	2	1	0	1	2	3
Age (years)	-	-	-	<45	45-54	55-64	>65
Heart rate (beats per minute)	-	<40	41-50	51-100	101-110	111-129	>130
Systolic pressure (mm hg)	<70	71-80	81-100	101-199	-	>200	-
Respiratory rate (breath per minute)	-	<9	-	9-14	15-20	21-29	>30
Temperature (degree celcius)	-	<35	-	35.0-38.4	-	>38.5	-
Consciousness	-	-	-	Clear	Response to voice	Response to pain	No response
Oxygen saturation (in percentage)	-	-	-	-	-	<90	<85

In the present study, the mean age was 58.22±15.52 years. The majority of the patients were older than 60 years of age. This is similar to the finding of the study conducted by Qin et al. where they observed that most of the patients were of elderly age [10]. In the present study, the mean heart rate in non-survivors was significantly higher than in the survivors. Kim et al. studied MEWS as a tool to predict for predicting in-hospital mortality in traumatic brain injury patients and observed that the non-survivors had significantly higher pulse rates as compared to the survivors [16]. Similarly, the non-survivors had significantly lower oxygen saturation in the present study as compared to the survivors. Qin et al. observed that the oxygen saturation in non-survivors and survivors was 92% and 95% respectively [10]. In our study, the oxygen saturation in non-survivors was significantly lower (88.29%) as compared to survivors (92.12%).

In the present study, the use of inotropes and vasopressors was observed to be significantly higher in non-survivors as compared to the survivors. Similar, findings were observed by Qin et al. in their study that the doses of inotropes and vasopressors administered were higher in non-survivors as compared to survivors [10].

In the present study, the AUROC for the new MEWS score in predicting mortality was 0.684 (p < 0.001), which was more than that of the MEWS score. Wang et al. conducted comparative research on the prognostic ability of APACHE II and MEWS and they observed that the latter had a better prediction of mortality as compared to the former. The area under the ROC curve for the APACHE score (0.79) was statistically significant compared to MEWS [17].

The ROC curves demonstrated that the new MEWS scoring system had a higher prediction of mortality than the old MEWS scoring system and a marginally lower prediction of mortality than the APACHE II and SOFA scores. In this study, we found that though the new MEWS score was better in predicting mortality when compared to the MEWS score, it was still inferior when compared with the SOFA score and APACHE II score. Huang et al. conducted a study for evaluating the scoring systems in trauma patients and observed that MODS warning scores had statistically significant ROC curve analysis as compared to SOFA and APACHE II scores [18]. However, they didn’t evaluate MEWS or new MEWS scores in their study. Qin et al. also observed better prediction of the new MEWS score on mortality of ICU admitted patients as compared to the MEWS score [10].

One of the limitations of the study is the use of new MEWS as a tool for evaluating the prognosis of rapidly evolving shock patients has yet to be implemented in the Indian scenario (due to the paucity of literature available), despite the fact that it is necessary to ensure effective and prudent use of our overburdened acute medical services and to reduce negative outcomes.

## Conclusions

Shock is a serious life-threatening condition and it is imperative that we should identify and treat it before it progresses to an irreversible stage. A number of scoring schemes have been developed to gauge the severity and mortality of shock. The present study advocates that the new MEWS score is better than the MEWS score in predicting mortality. The new MEWS score is a simple, entirely clinical, inexpensive scoring system that can be done within a short time of patient arrival to the casualty or critical care unit. Hence, the new MEWS score can help in the quick identification of patients with shock and can be used as a predictor of mortality.
